# "BECAUSE HE'S MY BABY": HOW MOTHERS EXPLAIN FAVORING THEIR FIRST- AND LAST-BORN CHILDREN IN LATER LIFE

**DOI:** 10.1093/geroni/igac059.2327

**Published:** 2022-12-20

**Authors:** Reilly Kincaid, J Jill Suitor, Megan Gilligan, Destiny Ogle

**Affiliations:** Purdue University, West Lafayette, Indiana, United States; Purdue University, West Lafayette, Indiana, United States; Iowa State University, Ames, Iowa, United States; Purdue University, West Lafayette, Indiana, United States

## Abstract

Past research suggests that adult children’s birth order is an important predictor of mothers’ favoritism in later life. Older mothers are especially likely to favor their last-born children for socioemotional relationship dimensions such as emotional closeness. However, researchers have not examined how children’s birth order affects the reasons underlying these patterns. To address this question, we examine: (1) how older mothers' reasons for favoritism differ by favored children’s birth order, and (2) how these differences help explain mothers' disproportionate likelihood of favoring their last-borns. Qualitative data were collected during in-home interviews with 156 older mothers as part of the Within-Family Differences Study-II. Ninety-two of the mothers chose their last-borns and 64 chose their first-borns as the children to whom they felt most emotionally close. Findings suggest that last-borns were most often favored because they were seen as understanding and empathetic or in greater need of mothers’ attention and support. First-borns were often favored based on birth order and geographical proximity or contact frequency. Birth order itself was mentioned in 17.39% and 23.44% of cases of favoritism toward last-borns and first-borns, respectively. These findings enrich and extend past research on the role of birth order in shaping intergenerational relationships by shedding light on the reasons why last-borns are often mothers’ emotionally closest children. Given that poor parent–child relationship quality is linked to worse physical and psychological health for both adult children and their older parents, findings have important implications for research on family well-being in later life.

